# Complete Genome Sequences of Phages EarickHC, Figueroism, FinalFrontier, SBlackberry, Skylord, and Slay isolated using *Microbacterium foliorum*

**DOI:** 10.17912/micropub.biology.001357

**Published:** 2024-12-02

**Authors:** Brianna Barruga, Sabrina Benitez, Emily Bouit, Tristan Bouit, Earick Cagang, Joshua Cantos, Dylan Carpio, Jaden Chen, Sungyoung Choi, Rita Dementyev, Ethan Dewri, Christian Dominguez, Alexandria Falvo, Christian Figueroa, Elva Garcia, Yannik Gibson, Dulce Guevara, Katie Jang, Michael Kelly, Caleb Kim, Nicole Kim, Raymond Kim, Julia Ko, Alyssa Lee, Yumin Lee, Rosalia Marenco, Moses Milano, Soojeong Moon, Kristen Ngo, Brian Nguyen, Eddie Nguyen, Ashley Perdomo, Elizabeth Paul, Ester Peiro, Daphne Prakash, Jennifer Ramon, Leslie Rodriguez, Emily Sandoval Plouffe, Joanna Shelim, Elizabeth Ton, Adam Tsao, Tabitha Usery, Madyson Vaca, Ethan Wang, Stuart Wettstein, Noboyuki Yano, Grace Young, Jiacheng Zhang, Arturo Diaz

**Affiliations:** 1 Biology, La Sierra University

## Abstract

Phages EarickHC, Figueroism, FinalFrontier, SBlackberry, Skylord, and Slay were isolated from soil samples collected around Southern California using the host *Microbacterium foliorum .* All six phages are lytic and have a siphoviral morphology. Genomes are 39,843 to 52,992 bp in length and contain 58 to 91 protein coding genes.

**Table 1. Phage genome information.** Genome characteristics of phages isolated using host *Microbacterium foliorum* NRRL B-24224, including GC content, genome length, character of genome ends, number of predicted open reading frames (ORFs), and number of predicted tRNAs. Phages were assigned to clusters based on gene content similarity of at least 35% to phages in the Actinobacteriophage database. Sequencing information for each phage as well as sample collection location and the phage isolation method are also listed.

**Table:** 

Phage	EarickHC	SBlackberry	Skylord	FinalFrontier	Slay	Figueroism
Sample location (city, state)	Riverside, CA	Eastvale, CA	Rancho Cucamonga, CA	Riverside, CA	Riverside, CA	Los Angeles, CA
GPS coordinates	33.910113 N, 117.51028 W	33.969167 N, 117.574444 W	34.15 N, 117.52 W	33.907829 N, 117.498698 W	33.90975 N, 117.501139 W	34.127029 N, 118.19046 W
Isolation method	Enriched	Direct	Enriched	Direct	Enriched	Enriched
Approximate read coverage (fold, x)	29	463	1720	1449	1161	7598
# of 150 base single-end reads	15,660	134,514	472,293	426,989	308,455	2,231,032
Genome length (bp)	52,992	42,048	39,843	42,046	41,922	41,847
GC content %	68.90%	66.90%	67.10%	66.50%	66.80%	63.50%
Character of genome ends	Circularly permuted	Circularly permuted	3’ sticky overhang	3’ sticky overhang	3’ sticky overhang	Circularly permuted
# of ORFs (# with predicted function)	91 (30)	58 (29)	70 (31)	70 (34)	71 (21)	63 (25)
# of tRNAs	0	0	2	1	1	0
Cluster Assignment	EC	EJ	EB	EB	EB	EA1
GenBank accession #	OP068338	MZ747515	OK999981	OQ995434	OQ995435	OP021683
SRA accession #	SRX20630263	SRX10050393	SRX10050394	SRX20630265	SRX20630258	SRX20630264

## Description

*Microbacterium* species are Gram-positive bacteria that are ubiquitous in nature, particularly in plants, soil, aquatic environments, and dairy products (Russel et al. 2019) . Various members of this genus, including *M. foliorum* and *M. paraoxydans* , have been detected in clinical samples such as wound swabs and blood specimens (Laffineur et al. 2003; Gneiding et al. 2008) . To expand our understanding of phage diversity and contribute to a growing collection of phages that infect *Microbacterium,* six phages were isolated from soil samples collected in Southern California using *Microbacterium foliorum* NRRL B-24224 as the host bacteria (Table 1) (Jacobs-Sera et al *.* 2020).

Soil samples were resuspended in peptone-yeast extract-calcium (PYCa) liquid medium and incubated at 30°C with shaking for 90 minutes. Samples were centrifuged and the supernatants were passed through a 0.22 μm filter (Poxleitner et al. 2018) . For phages discovered through a “direct” isolation method, the filtrate was plated in PYCa soft agar containing *M. foliorum* and incubated at 30°C for up to 48 h for plaque formation. For phages isolated through an “enriched” method, *M. foliorum* cells were added to the filtrates, and the samples were incubated at 30°C for 72 h with shaking. The samples were then refiltered before being plated on PYCa soft agar containing *M. foliorum* . All phages produced clear plaques, and were plaque-purified through three rounds of plating. High-titer lysates were used for negative-staining (uranyl acetate) transmission electron microscopy (TEM) (Poxleitner et al. 2018) and the phages were determined to have a siphoviral morphology (Table 1).

DNA was isolated from high-titer phage lysates using the Promega Wizard DNA Cleanup Kit. Genomic DNA was sequenced using an Illumina MiSeq sequencer (v3 reagents) after preparing individual libraries using the NEBNext Ultra II FS Kit. The number and approximate coverage of single-end 150 base reads are listed in Table 1. Raw reads were assembled into a single contig for each genome using Newbler v2.9 with default parameters (Russell 2018) . Genomes were visually checked for completeness using Consed v29 (Gordon and Green 2013) . The resulting genomes were 39,843-52,992 bp in length, with a GC content of 63.5-68.9 % (Table 1). Phages Skylord, FinalFrontier, and Slay contain 10-11 base 3'-sticky overhangs. In contrast, the genomes of SBlackberry, EarickHC, and Figueroism are circularly permuted (Table 1).

Phage Evidence Collection and Annotation Network (PECAAN) was used to annotate the genomes ( https://blog.kbrinsgd.org/overview/ ). Phage gene starts were predicted by Genemark v2.5 (Besemer and Borodovsky 2005) , Glimmer v3.02 (Delcher et al. 2007) , and Starterator v.546f. Transmembrane helices were predicted using DeepTMHMM v1.0.39 ( https://dtu.biolib.com/DeepTMHMM ) (Hallgren et al. 2022) , TOPCONS v2.0 ( https://topcons.cbr.su.se/pred/ ) (Tsirigos et al. 2015) , and SOSUI v1.11 ( https://harrier.nagahama-i-bio.ac.jp/sosui/mobile/ ) (Hirokawa et al. 1998) . tRNAs were detected using ARAGON v1.2.38 and tRNAscan-SE v2.0 (Laslett and Canback 2004; Lowe and Chan 2016) . Protein functions were determined using HHpred (PDB_mmCIF70, UniProt, Pfam-A v.36, and NCBI Conserved Domain databases) (Söding et al. 2005), BLASTp v.2.14.1 (Altschul et al. 1997) alignments against the Actinobacteriophage protein (Russell and Hatfull 2017) and NCBI non-redundant protein sequences databases (https://blast.ncbi.nlm.nih.gov), and Phamerator (Cresawan et al. 2011). Phages were assigned to clusters (Table 1) based on at least 35 % gene content similarity (GCS) to sequences in the Actinobacteriophage database, phagesDB, using the phagesDB GCS tool (Russell and Hatfull 2017) . All software were used with default parameters.

For the most part, the characteristics of the six phages described in this study support previous descriptions for their respective clusters (Jacobs-Sera et al *.* 2020; Kim et al *.* 2022). Notably, EarickHC contains an asymmetric sequence motif that is found repeated across the genomes of cluster EC phages; SBlackberry encodes a small number of genes of unknown function that interrupt the structural gene operon of cluster EJ phages; similarly, FinalFrontier, Slay and Skylord encode 3 - 4 genes distributed across each genome that interrupt the unidirectional transcription of all other genes in cluster EB phages; Figueroism encodes its tail assembly chaperones from two separate genes typical of subcluster EA1 phages. Finally, no integrase or repressor functions could be identified across any of these six phages, suggesting that these phages do not undergo lysogeny and are thus predicted to be lytic phages.

**Nucleotide sequence accession numbers**

GenBank and Sequence Read Archive (SRA) accession numbers are provided in Table 1.
